# Analysis of Food Storage Stability of Biodegradable Containers Made of Pork Skin Gelatin Polymer with Walnut Shell Powder

**DOI:** 10.3390/polym14101940

**Published:** 2022-05-10

**Authors:** Chang-Hwan Jeong, Sol-Hee Lee, Hack-Youn Kim

**Affiliations:** Department of Animal Resources Science, Kongju National University, Yesan-Gun 32439, Korea; jch782@naver.com (C.-H.J.); chzh73@naver.com (S.-H.L.)

**Keywords:** animal by-product, biodegradable container, food storage stability, gelatin polymer, walnut shell

## Abstract

This study analyzes the food storage stability of biodegradable containers made of pork skin gelatin polymer. Packaging materials were prepared with different proportions of walnut shell powder, including 10% (W10), 20% (W20), and polyethylene packaging (PE) as a control. To analyze storage stability, parameters such as pH, thiobarbituric acid reactive substance (TBARS), volatile basic nitrogen (VBN), microbial population, and color were measured. The pH, yeast and mold, redness, and yellowness of W10 and W20 had no significant difference compared to those of PE in all storage periods (*p* > 0.05). The TBARS of W20 was shown to slowly increase compared to W10. The VBN concentration of W10 and W20 were significantly higher than that of PE in the first and second weeks, but there was no significant difference in the third week (*p* < 0.05). The total bacterial counts of W10 and W20 were significantly higher than that of PE during the first week (*p* < 0.05), but there was no significant difference thereafter (*p* > 0.05). The lightness values of W10 and W20 were significantly lower than that of PE in the second and third weeks (*p* < 0.05). These results indicated that biodegradable containers containing up to 20% walnut shell powder can substitute plastic packaging materials.

## 1. Introduction

The consumption of plastic has steadily increased with industrial development [1]. Moreover, as it is light in weight and allows the mass production of a desired form, plastic is being widely used in a variety of industrial fields [2]. Plastic is primarily used in the production of disposable packaging containers or short-lived products that are often discarded after a short period of time [3]. Although plastic waste should be dealt with through landfill or incineration, it may still induce soil pollution, marine pollution, and air pollution [4,5,6]. To reduce the consequent environmental pollution, international organizations such as the World Wildlife Fund (WWF) and various countries worldwide are seeking out materials that can substitute plastic [7].

To replace plastic containers, eco-friendly plastics are produced such as biodegradable and biomass containers. Biodegradable containers are degraded by the microorganisms in soil upon landfill and lead to not only less environmental pollution, but also faster degradation than plastic containers due to their low compression strength [8,9]. Currently, biodegradable containers using agro-based polymers or by-products such as gelatin, egg yolk, and soy protein are being studied [10,11,12]. Manufacturing biodegradable containers using by-products reduces material costs, but it has disadvantages of those additional processes and the price of the final product may increase [13]. However, from the perspective of sustainable development goals (SDGs), the reuse of these can increase economic value and environmental benefits. 

Gelatin is a type of derived protein produced upon the hydrolysis of collagen found in animal bones, cartilage, and skin [14]. Gelatin shows high degrees of stickiness and tackiness during gelation, whereas the hardness increases after gelation with reduced stickiness and tackiness, which allows the form to be maintained [15]. The walnut shell has received growing attention as a material of lignocellulosic activated carbon that can reduce microbial contamination [16]. Activated carbon is a material that allows the adherence of contaminant molecules to its surface, allowing for the removal of contaminating substances [17]. In addition, the phosphorous (P) and potassium (K) contained in walnut shells could enrich the soil upon landfill [18].

Food contamination may occur during the storage and distribution based on the water or oxygen vapor transmission rate of the container packaging material [19,20]. Although foods are sterilized during production, the contamination caused by the packaging material could affect the shelf life [21]. Based on the Korean Ministry of Agriculture, most meat products are determined to be contaminated with a total bacteria count (TBC) of ≥ log 10^7^ colony-forming units (CFUs)/g, thiobarbituric acid reactive substances (TBARS) with ≥2.0 mg malondialdehyde (MDA)/kg, and volatile basic nitrogen (VBN) of ≥20 mg% [22,23,24]. In addition, as the packaging material has an effect on food, which could ultimately influence what consumers take from the food, it is essential to have a test of food storage stability on biodegradable containers after production [25]. Accordingly, research using bioactive substances based on agro-by-products such as phosvitin from egg yolk is being studied [26].

In previous studies, biodegradable containers have been produced using gelatin and walnut shells as plastic substituents, where the production ratio for walnut shell materials was set at two types [27]. In this study, therefore, the pH, TBARS, VBN, microbial population, and color were compared between a container of plastic packaging and biodegradable containers produced based on the two production ratios, with the aim of determining whether the so-produced biodegradable containers could substitute plastic packaging containers.

## 2. Materials and Methods

### 2.1. Production of Biodegradable Containers 

The biodegradable container was prepared according to [27]. Gelatin powder, eggshell powder, and walnut shell powder used in the preparation were purchased from Jewon International (Seoul, Korea), Ganong Bio (Pocheon, Korea), and The Witch (Seoul, Korea), respectively, with a particle size of 180 mesh at this time. Gelatin powder and distilled water were heated and mixed in a water bath (JSWB-30T; JSR, Chungnam, Korea) set to a temperature of 100 °C in a volume ratio of 1:3, and an eggshell was added as a pore material with 20% of the mixture. Then, walnut shell powders were added in volume ratios of 10% and 20% of the mixture, separately. After the mixture was hardened at 5 °C for 30 min, the diameter (Ø) and the height were set to 4.0 and 0.5 cm, respectively, and the molded containers were dried in a drying oven (C-F03; Vision Scientific, Daejeon, Korea) at 30 °C for 24 h, at 40 °C for 16 h, at 105 °C for 12 h, and at 121 °C for 15 min. Thereafter, 1% C_6_H_8_O_6_ and 1% C_6_H_10_CaO_6_ were sprayed on the containers as waterproof coating agents. The waterproof coated containers were dried at 30 °C for 24 h using a drying oven (C-F03; Vision Scientific) and used in the experiment.

### 2.2. Sausage Packaging and Storage Conditions

Vienna sausages sold by CJ CheilJeDang Corporation (Seoul, Korea) were purchased and used as a packaging material during sample storage. As for the packaging material, the polyethylene packaging material (PE) purchased from Onepack (Seoul, Korea) and biodegradable containers with 10% (W10) and 20% (W20) of walnut shell powder were used. The sausages were removed and bisected in a sterilized state in a biological safety cabinet (PCHC-777A2-05; CHC LAB, Daejeon, Korea). The W10 group and the W20 group were placed in such a way that the cross-sections of the samples were in contact with the biodegradable containers, and the same packaging material as the PE group surrounded the containers and the sausage surface. All groups were sealed with a vacuum packaging machine (C15HL; WEBOMATIC, Bochum, Germany) set at a vacuum of 20% and a temperature of 1.5 °C, and the packaged samples were refrigerated at 5 °C for 3 weeks and used in this study. 

### 2.3. pH

The pH was homogenized with 4 g of the sample and 16 mL of distilled water at 6451× *g* for 1 min using ultra turrax (HMZ-20DN; Pooglim Tech, Seongnam, Korea). The pH of the homogenous sample was measured using a pH meter (Model S220; Mettler-Toledo International, Greifensee, Switzerland) calibrated with buffer solutions (pH: 4.01, 7.0, and 10.0). 

### 2.4. TBARS

TBARS was measured by modifying the distillation method reported previously [28]. Here, 10 g of sample, 50 mL of distilled water, and 200 µL of 0.3% butylated hydroxytoluene (BHT) were homogenized using a homogenizer (AM-5; Nihonseiki Kaisha, Tokyo, Japan) at 5614× *g* for 2 min. Thereafter, the homogeneous solution, distilled water (47.5 mL), 4 N HCl (2.5 mL), boiling stone, and antifoaming agent were added to a flask and mixed. The flask was heated with a heating mantle (MS-E102; Lab Merchant, London, UK) at 100 °C to collect the distilled solution. Then, 5 mL of a distilled solution and 5 mL of 0.02 M 2-thiobarbituric acid in 90% acetate were mixed and heated in a water bath (JSWB-30T; JSR) at 100 °C for 35 min and cooled in cold water for 10 min. Subsequently, it was measured at 538 nm using a multi-mode microplate reader (Spectra Max ID3; Molecular Devices, San Jose, CA, USA). The standard used was 1,1,3,3-tetraethoxypropane (TMP) for the measurement, and the measured value was substituted in the standard curve and converted into the amount of MDA.

### 2.5. VBN

VBN was measured by modifying the Conway microdiffusion method [29]. Briefly, 10 g of sample and 20 mL of distilled water were homogenized using a homogenizer (AM-5; Nihonseiki Kaisha) at 5614× *g* for 1 min. The homogeneous solution was put in a 100 mL cylinder and made up to a mark with distilled water. Thereafter, the homogeneous solution was filtered with a filter paper (Whatman No. 1; Whatman, Maidstone, UK). Then 1 mL of filtrate was added to an outer Conway dish, and 1 mL of 0.01 N H_3_BO_3_ and 100 µL of Conway indicator (0.066% methyl red + 0.066% bromocresol green + ethanol) were added to an inner Conway dish. After that, 1 mL of 50% K_2_CO_3_ was added to the outer Conway dish, the lid covered with Vaseline was closed, and the reaction was carried out at 37 °C for 2 h. The equation for VBN content calculation was as follows:

$$VBN\;\left(mg\%\right)=\frac{\left(V_{1}-V_{2}\right)}{m}\times 0.14\times a\times b\times 100$$

where *V*_1_ is titration volume of sample solution; *V*_2_ is titration volume of blank; *m* is weight of sample; *a* is titer value of 0.02 N H_2_SO_4_; *b* is dilution factor.

### 2.6. Microbial Population

The microbial population was homogenized with a stomacher (WH4000-2751-9; 3M) of 0.1% buffer peptone water twice as much as the sample for 1 min. After collecting 1 mL of the mixed solution, it was diluted in 9 mL of 0.1% buffer peptone water, and the process was repeated as many times as necessary for dilution drainage. The TBC was confirmed using a tryptic soy agar badge (TSA), and *Escherichia coli* and yeast and mold (YM) were confirmed using 3M^TM^ Petrifilm (3M; Saint Paul, MN, USA). The homogeneous solution with a volume of 0.1 mL was dispensed in a TSA, then smeared and cultured at 37 °C for 24 h. One milliliter of *E. coli* and YM were dispensed, then smeared and cultured at 37 °C for 24 h and at 25 °C for 48 h. The colonies on the plates were counted and expressed as CFUs per gram of sample.

### 2.7. Color

The cross-section of the sample in contact with the packaging material was measured using a colorimeter (CR-10; Minolta, Tokyo, Japan), with an 8 mm measuring diameter and an 8 lx illumination angle (CIE standard illuminant D65). Color values are expressed as CIE L* (lightness), CIE a* (redness), and CIE b* (yellowness). The standard color was a white standard plate with a CIE L* of +97.83, a CIE a* of −0.43, and a CIE b* of +1.98.

### 2.8. Statistical Analysis

All analyses were conducted at least three times under each set of experimental conditions, and the mean value ± standard deviation of each parameter was determined. The quality properties and storage properties of the sausages stored in polyethylene packaging and biodegradable containers manufactured with walnut shell powder were analyzed by two-way ANOVA using the GLM procedure within the SAS (version 9.4 for windows; SAS Institute, Cary, NC, USA) program, which considers the additional level of the sample or storage period as a fixed effect and the batch as a random term. At a 5% significance level (*p* < 0.05), Duncan’s multiple range test was used to determine statistical differences among the sample means.

## 3. Results and Discussion

### 3.1. pH

Table 1 presents the pH values of sausages stored in the PE and biodegradable containers produced with walnut shell powder. Across all storage periods, the pH did not vary significantly according to the type of packaging material (*p* > 0.05). Food quality and safety are closely associated with pH, as microbial contamination causes a variation in pH [30]. However, no variation in pH was observed for the PE and biodegradable container groups, indicating that the biodegradable containers produced in this study had little effect on the pH of foods. In addition, the pH of the stored food is a critical factor in food product storage, because the direct contact between the packaging material and the packaged food could have a chemical effect, limiting the use of the packaging [31].

A trend of declining pH was observed over time in all experimental groups, with a significant drop in the third week of storage compared to in the second week of storage (*p* < 0.05). Microorganisms mediate glucose oxidation to gain ATP required for growth, and in this process, the pH decreases [32]. A fall in the pH of meat products below 5.0 was reported to have an effect on sensory properties such as taste and flavor due to the increase in acidity [33]. However, all experimental groups in this study showed a pH value of approximately 6.3 until the third week of storage, indicating minimal effects of reduced pH. Thus, the potential use of biodegradable containers produced with walnut shell powder to substitute PE has been verified with respect to pH.

### 3.2. TBARS

Table 2 presents the TBARS of sausages stored in the PE and biodegradable containers produced with walnut shell powder. Lipid oxidation may be accelerated in the presence of oxygen, and the accelerated lipid oxidation could increase the production of MDA to elevate the TBARS [34]. In addition, a packaging material with a high oxygen vapor transmission rate could increase the contact between oxygen and the meat product to have an effect on the TBARS [35]. Across all storage periods, the TBARS did not vary significantly according to the type of packaging material (*p* > 0.05). The lack of variation in the oxygen vapor transmission rate from the PE revealed that the biodegradable containers made with walnut shell powder had little effect on the food TBARS. With an increase in storage period, the TBARS showed a significant increase in the first week of storage for the W10 group and in the third week of storage for the W20 group compared to in the initial week (week zero) of storage (*p* < 0.05). The PE group, on the contrary, showed a significant increase in the second week of storage compared to in the initial week of storage (*p* < 0.05). Lipid oxidation occurs when oxygen reacts with unsaturated fatty acids, producing hydroperoxides [36]. The resulting compound is unstable and decomposes in time to generate secondary compounds such as aldehydes [37]. The slower increase in TBARS for the W20 group compared to for the W10 group is presumed to be due to the antioxidant activity of the walnut shell as the material of the biodegradable containers produced in this study. Reference [38] reported that the phenolic compounds in the walnut shell, including juglone and ellagitannins, demonstrate a high level of antioxidant activity in activated carbon based on the walnut shell. The correlation of the TBARS for the storage period was 99.9%, suggesting that the effects on storage were highly significant (*p* < 0.001). Based on the results, the potential use of biodegradable containers produced with walnut shell powder to substitute PE has been validated with respect to TBARS.

### 3.3. VBN

In the measurement of protein spoilage in line with microbial growth, the VBN may serve as an indicator of the meat product’s quality and shelf life [39]. Table 3 presents the VBN concentration of sausages stored in the PE and biodegradable containers produced with walnut shell powder. The VBN concentration was significantly higher for the W20 group than for the PE group in the first week of storage (*p* < 0.05), and it was significantly higher for the W10 group than for the PE group in the second week of storage (*p* < 0.05). However, in the third week of storage, no significant difference was found among the experimental groups, suggesting the little effect of the biodegradable containers produced with walnut shell powder on the meat product quality and shelf life. Across all experimental groups, the VBN concentration increased with the storage period and was significantly higher in the third week of storage than in the second week of storage (*p* < 0.05). However, as the VBN concentration in all experimental groups dropped below 20 mg% until the third week of storage, it was determined that the biodegradable containers made with walnut shell powder were able to maintain food storage stability for three weeks. According to [40], the VBN concentration is positively correlated with the counts of bacteria and the level of endogenous enzymes. This is due to the protein degradation mediated by the bacteria and the endogenous enzymes with an increase in storage period to produce low-molecular non-protein nitrogen species such as NH_3_, H_2_S, and CH_3_CH_2_S [41]. The correlations of the VBN concentration were 99% for the experimental groups and 99.9% for the storage period. Based on the results, the potential use of biodegradable containers produced with walnut shell powder to substitute PE has been verified with respect to VBN.

### 3.4. Microbial Population

Table 4 presents the microbial population of sausages stored in PE and biodegradable containers produced with walnut shell powder. The TBCs were significantly higher for the W10 and W20 groups than for the PE group in the first week of storage (*p* < 0.05), whereas no significant difference in TBC was found in the second and third weeks of storage (*p* < 0.05). Reference [42] reported the detection of microbial growth at Log3 CFU/g from sodium alginate (C_6_H_8_O_6_) used as the food-coating material. The higher TBC values for the W10 and W20 groups than for the PE group in the early stage of storage in this study are thus attributed to sodium alginate (C_6_H_8_O_6_) in the waterproof the coating serving as nutrients for microbial growth. In contrast, the lack of a significant difference in TBC in the second and third weeks of storage is presumed to be due to the antioxidant activity of the walnut shell material of the biodegradable containers. The microorganisms that can grow on foods vary according to pH. The initial pH of food samples used in this study was 6.61, a level that requires attention for the contamination by yeasts and fungi [43]. The lack of a significant difference in YM among the experimental groups across all storage periods indicated that the pH of sausages did not affected the microbial growth (*p* > 0.05). The presence of *E. coli* was not detected across all experimental groups, which is due to the secondary sterilization generally performed on the commercial processed meat products against the *E. coli* spores [44]. In addition, the Hazard Analysis Critical Control Point (HACCP) strictly regulates *E. coli* contamination of food products during distribution. As a result, biodegradable containers produced with walnut shell powder are not likely to influence *E. coli* contamination of food products during distribution. The microbial quality of meat products may be affected by various factors, including the animal slaughter process, product processing, storage temperature, and storage conditions [45]. The TBC measurement can, therefore, serve as an indicator of microbial spoilage. With an increase in storage period, the TBC also showed an increasing trend across all experimental groups. The maximum storage period for the PE and biodegradable containers produced with walnut shell powder is predicted to be up to two weeks, and the similar rates of microbial growth for the same length of period indicated the potential for distribution of the biodegradable containers produced in this study. According to [46], for Vienna sausage products manufactured in Korea, an oxygen absorbent is incorporated into the packaging material during production or the Modified Atmosphere Packaging (MAP) is applied. However, such additives or packaging techniques were not applied in the packing of sausages in biodegradable containers in this study, which presumably accounted for the faster rate of contamination than the general shelf life of three weeks for Vienna sausages. Further studies should investigate the food storage stability when including an oxygen absorbent in biodegradable containers produced with walnut shell powder. The results of this study have verified the potential use of biodegradable containers produced with walnut shell powder to substitute PE with respect to the microbial population.

### 3.5. Color

Table 5 presents the colors of sausages stored in the PE and biodegradable containers produced with walnut shell powder. In terms of lightness, significantly lower values were observed for the W10 and W20 groups than for the PE group in the second and third weeks of storage (*p* < 0.05). Thermoplastic materials such as PE exhibit a stable chemical structure based on the binding between carbon (C) and hydrogen (H) [47]. However, the chemical structure of the biodegradable containers produced in this study lacked stability due to the use of a mixture of animal and plant byproducts. The unstable structure caused the interior of the biodegradable containers to swell during the drying process, and it is presumed that water from the sample partially seeped into the interior. The W10 and W20 groups experienced a decrease in lightness, as the sample’s surface hardened. In addition, a material to improve the structural stability of the biodegradable containers made using walnut shell powder appears to be necessary, for which a follow-up study should be conducted. The correlation of lightness for the experimental groups, storage periods, and interactions between the two was 99.9%, suggesting that the effects of the samples, storage, and interactions between samples and storage were highly significant (*p* < 0.001). The redness and yellowness showed no significant variation for all experimental groups across all storage periods (*p* > 0.05). The lightness and redness showed a decreasing trend with an increase in storage period for all experimental groups, and the lightness had a significant fall in the first week of storage compared to in the initial week of storage (*p* < 0.05). Reference [48] reported that a fall in the redness of edible or red meat products was correlated with lipid oxidation. In this study, the increase in TBARS concentration in all experimental groups with an increase in storage period was thought to cause a fall in the redness. The yellowness showed a trend of increasing with an increase in storage period for all experimental groups. Reference [49] reported that a positive correlation was found between the storage period and the yellowness of meat products, which was under the influence of the greening caused by microbial growth. Thus, the findings of this study have verified the potential use of biodegradable containers produced with walnut shell powder as a replacement for PE with respect to the color.

## 4. Conclusions

Through an analysis of food storage stability in biodegradable containers made of pork skin gelatin polymer with walnut shell powder, we concluded the following: the storage characteristics such as pH and YM of the biodegradable container groups showed no difference in all the storage periods compared to those of the PE group. The TBARS of the biodegradable container groups did not show a difference in all storage periods compared to the PE group, but the W20 group showed a slow increase compared to that of the W10 group. In all, the VBN concentration and the TBC showed a higher value for the biodegradable container groups in the early stages of storage, but there was no difference in the third week of storage. As a visual feature that consumers prioritize when choosing food, the redness and yellowness of the biodegradable container groups did not show a difference in all samples compared to those of the PE group. Based on the above research results, the biodegradable containers manufactured with 20% walnut shell powder are expected to contribute to SDGs such as improving environmental pollution and increasing economic value by replacing commercially used plastic packaging.

## Figures and Tables

**Table 1:** pH values of sausages stored in the PE and biodegradable containers manufactured with walnut shell powder.

Trait	Storage Period (Weeks)	Samples			ANOVA ^4^
		PE ^1^	W10 ^2^	W20 ^3^	
pH	0	6.61 ± 0.05 ^A^	6.61 ± 0.05 ^A^	6.61 ± 0.05 ^A^	T^NS^ ^5^ S *** T·S^NS^
	1	6.56 ± 0.05 ^AB^	6.56 ± 0.04 ^B^	6.56 ± 0.05 ^AB^	
	2	6.51 ± 0.05 ^B^	6.50 ± 0.01 ^C^	6.51 ± 0.04 ^B^	
	3	6.33 ± 0.03 ^C^	6.35 ± 0.04 ^D^	6.35 ± 0.09 ^C^	

^1^ PE, polyethylene packaging. ^2^ W10, biodegradable container manufactured with 10% walnut shell powder. ^3^ W20, biodegradable container manufactured with 20% walnut shell powder. ^4^ ANOVA, two-way ANOVA analysis among the samples; T, sample; S, storage. ^5 NS^, non-significant (*p* > 0.05); ***, *p* < 0.001. All values are presented as the mean ± standard deviation. ^A–D^ The means in the same column with different letters are significantly different (*p* < 0.05).

**Table 2:** Thiobarbituric acid reactive substance (TBARS; mg malondialdehyde/kg sample) of sausages stored in the PE and biodegradable containers manufactured with walnut shell powder.

Trait	Storage Period (Weeks)	Samples			ANOVA ^4^
		PE ^1^	W10 ^2^	W20 ^3^	
TBARS	0	0.66 ± 0.04 ^B^	0.66 ± 0.04 ^C^	0.66 ± 0.04 ^B^	T^NS^ ^5^ S *** T·S^NS^
	1	0.68 ± 0.05 ^B^	0.74 ± 0.04 ^B^	0.72 ± 0.05 ^B^	
	2	0.80 ± 0.07 ^A^	0.76 ± 0.02 ^B^	0.74 ± 0.07 ^B^	
	3	0.82 ± 0.04 ^A^	0.93 ± 0.03 ^A^	0.87 ± 0.11 ^A^	

^1^ PE, polyethylene packaging. ^2^ W10, biodegradable container manufactured with 10% walnut shell powder. ^3^ W20, biodegradable container manufactured with 20% walnut shell powder. ^4^ ANOVA, two-way ANOVA analysis among the samples; T, sample; S, storage. ^5 NS^, non-significant (*p* > 0.05); ***, *p* < 0.001. All values are presented as the mean ± standard deviation. ^A–C^ The means in the same column with different letters are significantly different (*p* < 0.05).

**Table 3:** Volatile basic nitrogen concentration (mg%) of sausages stored in the PE and biodegradable containers manufactured with walnut shell powder.

Trait	Storage Period (Weeks)	Samples			ANOVA ^4^
		PE ^1^	W10 ^2^	W20 ^3^	
VBN	0	0.70 ± 0.16 ^C^	0.70 ± 0.16 ^D^	0.70 ± 0.16 ^C^	T **^5^ S *** T·S^NS^
	1	0.93 ± 0.32 ^BCb^	1.49 ± 0.32 ^Cab^	1.54 ± 0.28 ^Ba^	
	2	1.40 ± 0.28 ^Bb^	1.96 ± 0.28 ^Ba^	1.49 ± 0.16 ^Bab^	
	3	4.62 ± 0.48 ^A^	4.69 ± 0.27 ^A^	4.48 ± 0.23 ^A^	

^1^ PE, polyethylene packaging. ^2^ W10, biodegradable container manufactured with 10% walnut shell powder. ^3^ W20, biodegradable container manufactured with 20% walnut shell powder. ^4^ ANOVA, two-way ANOVA analysis among the samples; T, sample; S, storage. ^5^ ^NS^, non-significant (*p* > 0.05); **, *p* < 0.01; ***, *p* < 0.001. All values are presented as the mean ± standard deviation. ^a,b^ The means in the same row with different letters are significantly different (*p* < 0.05). ^A–D^ The means in the same column with different letters are significantly different (*p* < 0.05).

**Table 4:** Microbial populations (log colony-forming unit/kg) of sausages stored in the PE and biodegradable containers manufactured with walnut shell powder.

Trait	Storage Period (Weeks)	Samples			ANOVA ^4^
		PE ^1^	W10 ^2^	W20 ^3^	
TBC ^6^	0	3.25 ± 0.16 ^C^	3.25 ± 0.16 ^D^	3.25 ± 0.16 ^D^	T^NS 5^ S *** T·S^NS^
	1	3.66 ± 0.43 ^Cb^	4.66 ± 0.06 ^Ca^	4.29 ± 0.54 ^Ca^	
	2	6.44 ± 0.14 ^B^	6.72 ± 0.20 ^B^	6.45 ± 0.29 ^B^	
	3	8.73 ± 0.02 ^A^	8.82 ± 0.25 ^A^	8.89 ± 0.25 ^A^	
YM ^7^	0	1.68 ± 0.35 ^B^	1.68 ± 0.35 ^B^	1.68 ± 0.35 ^C^	T^NS^ S *** T·S^NS^
	1	2.98 ± 0.12 ^A^	2.93 ± 0.13 ^A^	2.77 ± 0.20 ^B^	
	2	3.25 ± 0.51 ^A^	3.18 ± 0.69 ^A^	3.16 ± 0.50 ^AB^	
	3	3.54 ± 0.31 ^A^	3.55 ± 0.25 ^A^	3.59 ± 0.34 ^A^	
*E. coli*	0	Non detection			
	1				
	2				
	3				

^1^ PE, polyethylene packaging. ^2^ W10, biodegradable container manufactured with 10% walnut shell powder. ^3^ W20, biodegradable container manufactured with 20% walnut shell powder. ^4^ ANOVA, two-way ANOVA analysis among the samples; T, sample; S, storage. ^5 NS^, non-significant (*p* > 0.05); ***, *p* < 0.001. ^6^ TBC, total bacterial count. ^7^ YM, yeast and mold. All values are presented as the mean ± standard deviation. ^a,b^ The means in the same row with different letters are significantly different (*p* < 0.05). ^A–D^ The means on the same column with different letters are significantly different (*p* < 0.05).

**Table 5:** Color of sausages stored in the PE and biodegradable container manufactured with walnut shell powder.

Trait	Storage Period (Weeks)	Samples			ANOVA ^4^
		PE ^1^	W10 ^2^	W20 ^3^	
L*	0	66.90 ± 1.06 ^A^	66.90 ± 1.06 ^A^	66.90 ± 1.06 ^A^	T ***^5^ S *** T·S ***
	1	65.33 ± 0.98 ^B^	65.20 ± 0.62 ^B^	65.75 ± 0.60 ^B^	
	2	64.33 ± 0.28 ^Ba^	61.70 ± 0.26 ^Cb^	61.97 ± 0.39 ^Cb^	
	3	64.50 ± 0.46 ^Ba^	56.83 ± 1.44 ^Db^	58.24 ± 0.34 ^Db^	
a*	0	12.10 ± 0.36 ^A^	12.10 ± 0.36 ^A^	12.10 ± 0.36 ^A^	T^NS^ S *** T·S^NS^
	1	11.50 ± 0.37 ^B^	11.47 ± 0.51 ^AB^	11.28 ± 0.26 ^B^	
	2	11.10 ± 0.14 ^B^	10.87 ± 0.65 ^B^	10.65 ± 0.22 ^C^	
	3	10.50 ± 0.23 ^C^	10.50 ± 0.44 ^B^	10.53 ± 0.53 ^C^	
b*	0	7.83 ± 0.78 ^B^	7.83 ± 0.78 ^B^	7.83 ± 0.78 ^B^	T^NS^ S *** T·S^NS^
	1	9.45 ± 0.41 ^A^	9.38 ± 0.30 ^A^	9.30 ± 0.18 ^A^	
	2	9.87 ± 0.46 ^A^	9.68 ± 0.38 ^A^	9.78 ± 0.48 ^A^	
	3	10.10 ± 0.19 ^A^	9.65 ± 0.21 ^A^	9.93 ± 0.46 ^A^	

^1^ PE, polyethylene packaging. ^2^ W10, biodegradable container manufactured with 10% walnut shell powder. ^3^ W20, biodegradable container manufactured with 20% walnut shell powder. ^4^ ANOVA, two-way ANOVA analysis among the samples; T, samples; S, storage. ^5^ ^NS^, non-significant (*p* > 0.05); ***, *p* < 0.001. All values are presented as the mean ± standard deviation. ^a,b^ The means in the same row with different letters are significantly different (*p* < 0.05). ^A–D^ The means in the same column with different letters are significantly different (*p* < 0.05).

## Data Availability

Not applicable.
